# Breast Cancer Care Quality Indicators in Spain: A Systematic Review

**DOI:** 10.3390/ijerph18126411

**Published:** 2021-06-13

**Authors:** Marta Maes-Carballo, Yolanda Gómez-Fandiño, Carlos Roberto Estrada-López, Ayla Reinoso-Hermida, Khalid Saeed Khan, Manuel Martín-Díaz, Aurora Bueno-Cavanillas

**Affiliations:** 1 Department of General Surgery, Complexo Hospitalario de Ourense, 32005 Ourense, Spain; yfandino@gmail.com (Y.G.-F.); estradacarlos79@gmail.com (C.R.E.-L.); aylareinosohermida@gmail.com (A.R.-H.); 2 Department of Preventive Medicine and Public Health, University of Granada, 18016 Granada, Spain; profkkhan@gmail.com (K.S.K.); abueno@ugr.es (A.B.-C.); 3 Department of General Surgery, Hospital Público de Verín, 32600 Ourense, Spain; 4 CIBER of Epidemiology and Public Health (CIBERESP), 28029 Madrid, Spain; 5 Department of General Surgery, Hospital de Motril, 18600 Granada, Spain; vistamar7@gmail.com; 6 Instituto de Investigación Biosanitaria IBS, 18012 Granada, Spain

**Keywords:** breast cancer care, quality indicators, quality care, health care, Spanish quality care

## Abstract

Breast cancer (BC) management care requires an increment in quality. An initiative to improve the BC quality care is registered, and quality indicators (QIs) are studied. We appraised the appearance of QIs and their standards systematically in Spain. A prospective systematic search (Prospero no: CRD42021228867) for clinical pathways and integrated breast cancer care processes was conducted through databases and the World Wide Web in February 2021. Duplicate data extraction was performed with 98% reviewer agreement. Seventy-four QIs (QI per document mean: 11; standard deviation: 10.59) were found in 15 documents. The Catalonian document had the highest number of QIs (*n* = 30). No QI appeared in all the documents. There were 9/74 QIs covering structure (12.16%), 53/74 covering process (71.62%), and 12/74 covering outcome (16.22%). A total of 22/66 (33.33%) process and outcome QIs did not set a minimum standard of care. QIs related to primary care, patient satisfaction, and shared decision making were deficient. Most of the documents established a BC QI standard for compliance, but the high variability hinders the comparison of outcomes. Establishing a consensus-based set of QIs needs urgent attention.

## 1. Introduction

Technological advancement has improved the early detection and treatment of breast cancer (BC) and has enhanced overall survival [1]. Nowadays, BC care management is more intricate and requires an increment in quality. An initiative to improve the quality of BC care is registered, and quality indicators (QIs) are studied [2]. The EUSOMA (European Society of Breast Cancer Specialists) working group states that “BC QIs provide a set of metrics to allow centres to follow patients over time in a standardised manner, and easily recognise when attention is required to improve particular areas of healthcare delivery” [2]. These must be explained in quality documents for the standardisation of care as clinical pathways or integrated breast cancer care processes elaborated by official institutions [3,4,5]. There are three types of QIs [6]: indicators of structure (evaluates all the sources used during the provision of services), process (appraises the actions done during patient care), and outcome (studies the results of patient care) [7,8]. In recent years, patient-centred care and shared decision making (SDM) (i.e., “a communication process in which clinicians and patients work together to share the best available evidence, consider options, and reach decisions about care according to their choices and beliefs”) [9] have gained importance [10,11,12,13]. Thus, there should be QIs focused on the evaluation of SDM [14].

Numerous authorities have suggested their own sets of QIs to establish BC quality management evaluation, but no agreement has been reached [15]. For example, in Europe, EUSOMA [2] has published a compilation of QIs that could be embraced by breast centres to provide standardised auditing and quality support and to establish an acceptable minimum standard of care. In Spain, the BC organisation varies among the 16 autonomous communities. Basic services are respected in all of them, but they establish specific health plans and adapt resources to the needs of the assigned population [16]. Every autonomous community has its own document for BC care quality (clinical pathways or integrated breast cancer assistance processes). These are quality documents deployed to manage and standardise BC care for a well-defined group of patients during a period of time and establish structured criteria for quality of care. The variability of this type of documents makes comparisons of results across populations or hospitals difficult [3,17,18,19,20,21,22]. There is also no legal obligation or incentives to report BC care management in public health. Therefore, BC care quality data analysis is heterogeneous [23].

Our literature search found no reviews about BC management QIs in health administrations in Europe or Spain. We appraised the appearance of QIs and their standards of care in Spanish quality documents systematically, paying special attention to the particular populations to which they are directed and comparing them with those suggested by EUSOMA [2].

## 2. Methods

We identified studies through a systematic review of the literature following prospective registration (Prospero no: CRD42021228867) and reported according to the PRISMA statement (Preferred Reporting Items for Systematic Reviews and Meta-Analyses) [24,25].

### 2.1. Data Search and Selection

Eligible studies included clinical pathways and integrated health care processes from Spanish administrations. The research was performed without language limitations on online databases (Medline, Web of Science, Embase, and Scopus). The MeSH terms “breast cancer”, “breast neoplasms”, “quality indicators”, and “quality care” were combined with other word alternatives in February 2021. The search strategy appears in Appendix A. Clinical pathways and integrated health care processes are usually not promulgated in medical journals or indexed. A comprehensive manual search of grey literature was conducted to find these BC quality documents elaborated by Spanish institutions on the World Wide Web. We also explored the bibliographies of the papers added to incorporate other crucial studies to our analysis.

### 2.2. Study Selection and Data Extraction

Three reviewers (YGF, ARH, and CREL) independently selected studies for inclusion in the review. The inclusion criteria were integrated breast cancer care processes and clinical pathways provided by Spanish national institutions. These are quality documents disposed to guide and standardise BC care for a well-defined group of patients during a period of time [26] and set structured criteria for quality of care [27]. We only collected documents that explicitly mentioned BC in a section of writing. We rejected observational studies, narrative reviews, scientific reports, discussion papers, conference abstracts and posters, randomised controlled trials (RCTs), clinical practice guidelines, and consensus. Full-text versions of conceivably relevant citations were obtained to confirm acceptability. A fourth reviewer (MMC) assisted in solving disagreements by consensus or arbitration. Where multiple versions were retrieved, the most updated version of the guidelines was incorporated. Duplicate articles were identified and deleted. We considered the EUSOMA working group’s position paper [2] as a reference to compare QIs. Data were extracted from the selected BC QI initiatives in duplicate and independently using standardised data extraction forms specifically created for this review and subsequently entered into a database. All data entry was double-checked.

### 2.3. Quality Assessment

The reporting of BC QIs from EUSOMA’s position paper [2], the Spanish integrated cancer care processes and clinical pathways, was independently appraised by three different reviewers (YGF, CREL, and ARH) using a piloted data extraction form. No suitable data extraction form was available for this research topic. We developed a descriptive quality scoring system that captured all the QIs and specified the document. These QIs collected in our review have already been validated in the quality documents (clinical pathways and integrated breast cancer care processes) where they belong. Disparities among the authors over the risk of bias for particular manuals were solved by group discussion, requiring a mediator (MMC) who decided when no consensus achieved. Two QIs were recognised as the same when they measured the same process, even when there were scanty differences between population targets and minimum standards. All these deviations were reported individually in the Results section of this paper. These studied QIs were classified according to the EUSOMA classification [2] concerning the intervention they were measuring (diagnosis, treatment, staging, counselling, follow-up, and rehabilitation) and Donabedian’s framework type (structural, process, and outcome indicators) [6].

### 2.4. Data Analysis

The inter-rater agreement (ICC) of the data extraction was calculated to assess the reviewers’ agreement, and ICC >0.90 was considered excellent [28]. A mediator (MMC) assisted in reaching a consensus and would decide in case of disagreements. We performed a descriptive statistical study to examine and classify the selected BC QIs using the Stata 15.0 statistical package (StataCorp LLC, College Station, TX, USA).

## 3. Results

### 3.1. Study Selection

**Figure 1:**
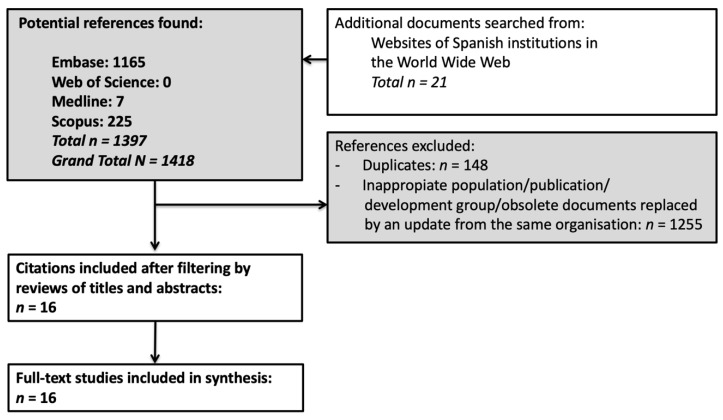
Flow chart of the literature search.

We identified 1418 relevant references (1165 from databases and 21 from the World Wide Web and Spanish institutions). Of them, 148 were duplicated reports, and 1255 did not satisfy the selection criteria. Finally, only 15 Spanish quality documents and the EUSOMA position paper were evaluated for full-text review [2,29,30,31,32,33,34,35,36,37,38,39,40,41,42]. A PRISMA flow diagram is synthesised in Figure 1. The study characteristics are reported in Table 1 (year of publication, organisation, region, evidence analysis used for QI evaluation, type of document (specific BC document or not), the presence of a specific section on BC, the appearance of QIs in the document analysed). Table 1 also shows four autonomous communities from Spain without a quality care document (Balearic and Canary Islands, Cantabria, and Castile and La Mancha).

### 3.2. General Quality Indicator Evaluation

**Figure 2:**
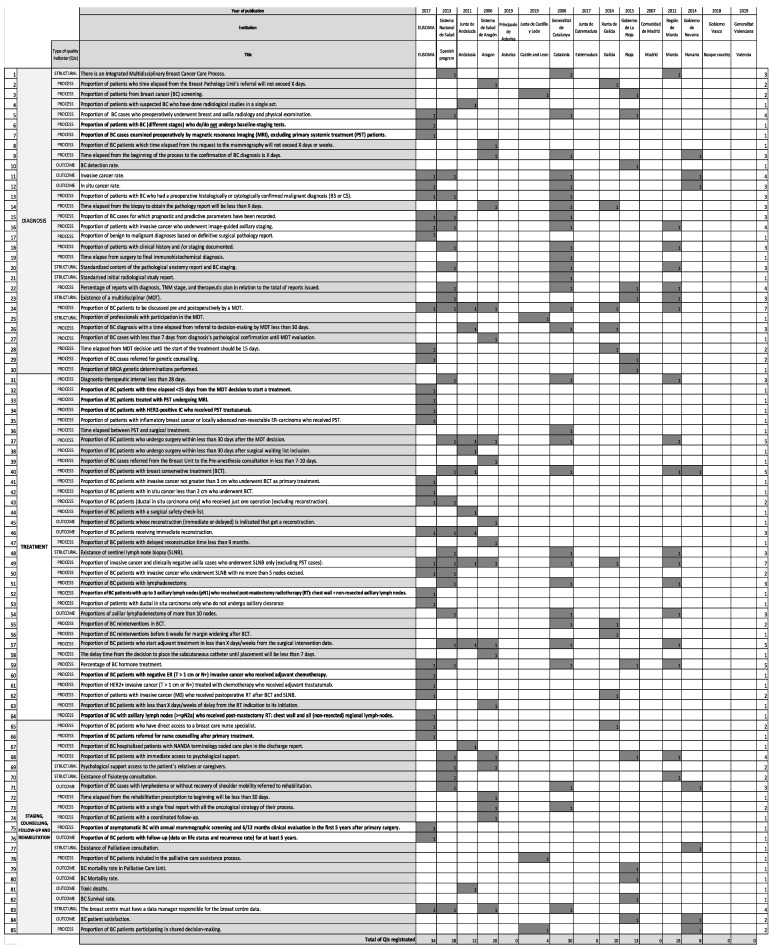
Appearance of the quality indicators (QIs) on diagnosis, staging, counselling, follow-up, and rehabilitation and others in the integrated BC health care process and clinical pathways analysed. QIs in bold were just published in EUSOMA. QIs in grey appeared in the Spanish documents analysed but not in the EUSOMA position paper.

There were 85 QIs collected from the quality care documents analysed. The EUSOMA position paper [2] registered 34/85 QIs (40%). The 51/85 (60%) QIs that did not appear in the EUSOMA position paper were added after a comprehensive analysis of the Spanish documents. Only 11/85 (12.94%) QIs appeared only in the EUSOMA paper. Figure 2 shows all the integrated health care programs and clinical pathways studied and the QIs appearing in them. From the Spanish documents, there were 28/74 QIs related to diagnosis (37.84%), the same number (28/74) related to treatment (37.84%), and 18/74 (24.32%) QIs to staging, counselling, follow-up, and rehabilitation. Nine of these Spanish QIs were structural (12.16%), 53/74 were related to the process (71.62%), and 12/74 were outcome QIs (16.22%). Analysing EUSOMA indicators that did not appear in any of the Spanish documents, 2 were related to diagnosis (18.18%), 6/11 related to treatment (54.54%), 1/11 to counselling (9.09%), and 2/11 to follow-up (18.18%). Inter-rater agreement was 0.98.

### 3.3. Quality Indicator Comparison between Spanish Areas and Europe

The BC QI reporting was varied (Figure 2). The QI mean in each document was 11.00 (standard deviation: 10.59), ranging from 0 to 30 QIs reported. The clinical pathways or integrated breast cancer care processes that collected more QIs were EUSOMA’s [2] with 34 QIs, Catalonia’s [34] with 30 QIs, and the government of Spain´s [29] with 28 QIs. Asturias [32], Extremadura [35], Madrid [38], Basque Country [41], and Valencia [42] did not register any QI.

Comparing the Spanish quality documents and the EUSOMA position paper [2], all the clinical pathways and integrated cancer care processes that collected any BC QI had at least one EUSOMA QI included. The national Spanish program [29] was the document that collected more EUSOMA QIs (12 QIs), followed by the Catalonian program [34] with 10 QIs.

No indicator appeared in all the 16 documents studied. Of the 51 indicators that appeared only in the Spanish documents, “proportion of BC patients to be discussed pre- and postoperatively by a multidisciplinary team (MDT)” and “proportion of invasive cancer and clinically negative axilla cases who underwent sentinel lymph node biopsy (SLNB) only (excluding primary systemic treatment or PST cases)” were the two QIs best reported, appearing in up to 6/15 different documents [29,30,31,34,36,39].

**Figure 3:**
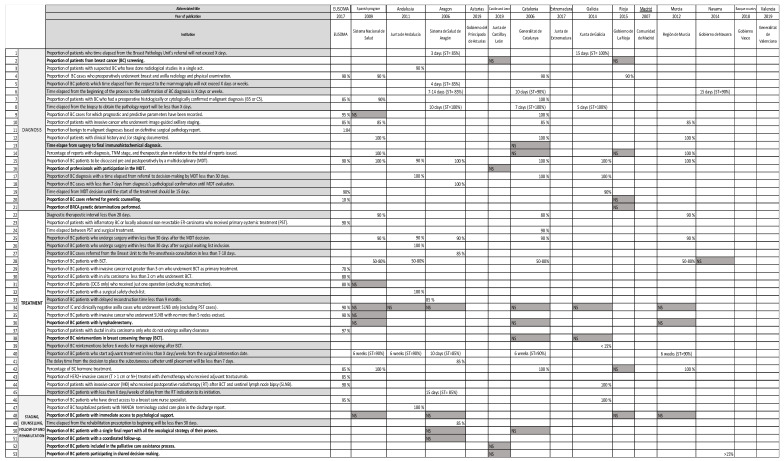
Comparison of the standards of the breast cancer care quality indicators related to the process among the Spanish integrated breast cancer health care processes and clinical pathways analysed. NS in grey means “standard not specified”.

**Figure 4:**
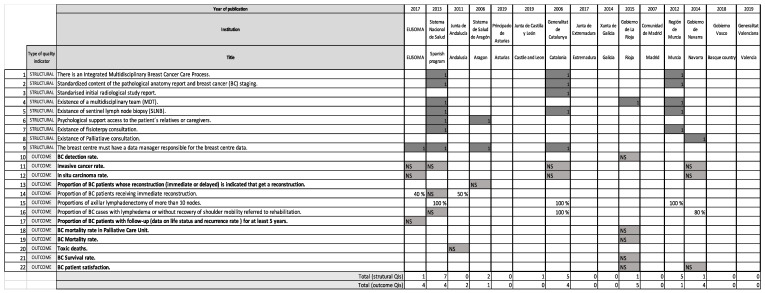
Quality indicators of structure and outcome in the Spanish breast cancer integrated health care processes and clinical pathways analysed.

The variability of the same QI among the diverse Spanish papers analysed is registered in Figure 3 and Figure 4. A total of 22/66 (33.33%) process and outcome QIs (12/53; 22.64% related to the process and 10/13; 76.92% outcome QIs) did not express any standard (Figure 3); the structure indicators do not present standards.

Concerning diagnosis, “proportion of BC cases who preoperatively underwent breast and axilla radiology and physical examination” appeared in three documents [29,34,37] that agree with a standard of 90%. “Proportion of BC cases for which prognostic and predictive parameters have been recorded” should be more than 100% [34], compared with EUSOMA’s [2] recommendation of 95%. “Proportion of patients with invasive cancer who underwent image-guided axillary staging” should be in all the cases at more than 85% [2,29,34,39], while “proportion of patients with clinical history and/or staging documented” might be 100% [29,34,39]. “Proportion of BC patients to be discussed pre- and postoperatively by a MDT” varied from 90% recommended by EUSOMA [2] and Andalusia [30] to 100% supported by the Spanish national document [29], Aragon [31], Catalonia [34], Galicia [36], and Murcia [39].

Regarding treatment, “proportion of BC patients with breast-conserving therapy (BCT)” did not arise in EUSOMA, but it was treated in a third part of the Spanish quality care papers (5/15). All of these documents except one [29,30,34,39] stated a standard of 50–80% [40]. The “percentage of BC hormone treatment” standard was always 100% in the Spanish documents [29,34,39], but 85% in the EUSOMA position paper. “All the patients with invasive cancer (M0) who received postoperative radiotherapy after breast-conserving surgery and SLNB” might be 100% [36], in contrast with only 90% in EUSOMA [2].

Analysing outcome QIs, the “proportion of BC patients receiving immediate reconstruction” standard was more than 50% in Andalusia [39] versus 40% in EUSOMA [2]. Finally, “proportion of BC cases with lymphedema or without recovery of shoulder mobility referred to rehabilitation” should be 80% in Navarra [40] versus 100% in Catalonia [34].

### 3.4. Shared Decision Making as an Essential Quality Indicator

We studied the appearance of SDM in the integrated cancer care processes and clinical pathways analysed. Only Castile and Leon [33] and Navarra [40] admitted its importance (see Figure 2). Navarra highlighted the importance of involving at least 15% of the patients in the BC care management decision. No other QIs about SDM use or measures were found.

### 3.5. Quality Indicators about Timing Processes

Figure 3 refers to all the indicators about timing in grey, followed by the standard established by the different quality care documents. Some of them are noteworthy in the following text. The QIs not mentioned are analysed in Figure 4. There were 18 QIs about the timing process, and only 1 (0.05%) did not state any standard.

Concerning diagnosis, “proportion of patients who time-elapsed from the breast pathology unit’s referral should not exceed 3 days [31] or 15 days” [36] depending on the quality care document with a standard that varied from 85% to 100%. The “time elapsed from the beginning of the process to the confirmation of BC diagnosis should be 7–14 or 10 days” standard varied from 90% or 85%, respectively [31,36]. “Time elapsed from the biopsy to obtain the pathology report will be less than 5 [36], 7 [34], or 10 [31] days”, and the “BC diagnosis should be referral to MDT in less than 30 days” [30,34,36] in both cases with a standard of 100%.

Regarding treatment, the “diagnostic–therapeutic interval must be less than 28 days” in more than 80% [34] to 90% [29,39] of the BC patients. The “proportion of BC patients who undergo surgery within less than 30 days after the MDT decision” QI, although it did not appear in EUSOMA, reached the highest consensus with a five-document agreement standard of 90% [29,30,31,34,39]. Finally, the “proportion of BC patients who start adjuvant treatment in less than a specific date from the surgical intervention date” QI had an enormous variability. Four quality care documents’ [29,30,34,39] standard was 90% in 6 weeks, but Aragon’s [31] clinical pathway stated 85% in 10 days.

## 4. Discussion

### 4.1. Main Findings

No systematic reviews were found in our search for Spanish health care QIs collected from integrated health care processes or clinical pathways. Only one-tenth of the indicators appeared exclusively in EUSOMA [2], including only 4 out of 10 of the QIs identified. There was heterogeneity among the QIs. No single indicator appeared in all the documents studied, and there was an enormous variability in QI descriptions. Over three-quarters were QIs dedicated to diagnosis and treatment, and the majority were process related. The QIs collected mostly were “proportion of BC patients to be discussed pre- and postoperatively by an MDT” and “proportion of invasive cancer and clinically negative axilla cases who underwent SLNB only (excluding PST cases)”. A third of the process and outcome QIs did not state a standard for reference.

### 4.2. Strengths and Weaknesses

To our knowledge, a collation of BC care management QIs has not been published before. We undertook a comprehensive systematic review with many expert reviewers studying an important number of integrated BC assistance processes and clinical pathways. This review provided a powerful insight into the state of QIs for the whole BC care management process, including diagnosis, treatment, and follow-up.

The data extraction’s subjective character was addressed using three qualified and trained BC specialist clinicians. The reviewers held a consensus meeting to consolidate criteria before duplicate data extraction appraisal. A fourth reviewer arbitrated the work to get a consensus when a meaningful deviation among the reviewers appeared. The ICC was higher than 98%, denoting an excellent reviewer agreement.

A possible limitation was to compare the Spanish clinical pathways or integrated breast cancer assistance processes versus the EUSOMA position paper [2]. The Spanish documents covered all the phases required in the BC care management process, from the general practitioner’s referral to the follow-up, while the European document was directed to the specific BC unit of care. However, this could be considered an advantage as including these Spanish manuals has shown the necessity for adding all levels and aspects of care in BC quality assessment.

One limitation could be geographical in that only Spanish documents were assessed in this review. However, our main objective was to highlight the level of consensus when choosing QIs of an important disease such as BC in the same country. Our findings emphasised the importance and urgent need for agreement about this issue. A strong point of this systematic review is that our team included researchers competent in both the English and Spanish languages. There was no need to use external translations to interpret any report.

Most of the studied papers were not academic articles in scientific journals or indexed in databases. Although it was not easy, a comprehensive manual search of grey literature was conducted to find administrations and official institutions engaged in BC care management quality on the World Wide Web. We engaged expert reviewers in this clinical field to ensure that we captured the totality of the relevant literature. We also searched in the identified publications’ bibliographies to incorporate more studies into our review. An interesting observation is that we did not find any document in order to analyse QIs in only three Spanish areas.

### 4.3. Implications

Our systematic review offers a crucial contribution to BC care quality assessment. It presents an extensive study of all the available BC care QIs in Spain and highlights relevant discrepancies among the studied integrated health care processes and clinical pathways. It provides a global overview of the current situation of the QIs by identifying areas in need of urgent improvement. Medical improvements are occurring quickly, so continuous development and periodic updates are needed. The BC care process’s control and progress could be made by analysing a single set of QIs and would help correlate results with other centres so stronger conclusions could be obtained [3].

The physical environment or structure is imperative in influencing the health care delivery process and outcomes. This physical environment will require supporting staff plus a healing environment to conclude better quality care finally [43].

Nowadays, even though diverse institutions have published different indicators to assess BC care quality, there is yet no consensus on BC QIs even in the same country [15,44]. Hence, correlation among studies is challenging, and this reduces the feasibility of comparing outcomes among different hospitals or health care areas [3]. Sometimes the same QIs could be interpreted as measuring different aspects of care [45]. Quality is a wide concept that needs a range of QIs to analyse various dimensions of care.

Even though only a few indicators have appeared exclusively in EUSOMA [2], it should be noted that the Spanish documents have not collected indicators about the use of magnetic resonance imaging in BC care, nurse counselling, and follow-up. These EUSOMA indicators should be reviewed and added to them in the next updates. On the other hand, the Spanish documents provided many indicators that EUSOMA did not collect, but no indicators were found about primary care or patient satisfaction. The European position paper [2] indicates that more studies are necessary to establish satisfaction indicators, but it does not consider indicators related to primary care. Obtaining QIs at all breast cancer care levels should be highlighted as an important point of improvement to control and improve cancer quality care and not only focus on breast units. All the links in the chain are important to obtain excellent results. Besides, SDM, a recognised pillar of high-quality cancer care, was vaguely included in only two documents. Forthcoming reviews should give deep consideration to primary care, patient satisfaction, and SDM.

A minimum standard of quality care is beneficial to evaluate compliance and the necessity for improvement. In this review, we found proposed standards for two-thirds of the process and outcome indicators, but there was high variability among documents. For example, most of the documents proposed that adjuvant treatment should start in 6 weeks in 90% of the patients, but only one document set 10 days in 85% of the patients [31]. Evidence indicates that the ideal time to start treatment is 4–8 weeks, permitting recovery from surgery. A longer delay could be associated with worse outcomes and increased mortality [46] due to the rapid growth of micrometastasis following the removal of the primary tumour [47]. Therefore, all the QIs should be evidence based.

Further research and consensus regarding the best BC QIs and standards for improving quality is needed and deserves immediate consideration. There is an urgent need for a compendium of common QIs and their standards of care for all the autonomous communities in Spain. Each region should design specific QIs, taking into account the particular characteristics of its population. Thus, a set of common and specific QIs should be developed to allow a homogeneous analysis of the BC quality of care and comparisons among regions.

## 5. Conclusions

There is no consensus concerning BC care QIs and standards in Spain, and QI focus on primary care, patient satisfaction, and SDM is lacking. Although a majority of the QIs established a standard, they were very varied. These differences made comparisons among different health care providers arduous, decreasing the chance of making reasonable comparisons. There is an urgent need for establishing an agreed set of BC care QIs. Common and specific QIs should be developed to allow a homogeneous analysis of the BC quality of care and comparisons among regions.

## Figures and Tables

**Table 1:** Characteristics of the clinical pathways and Spanish integrated BC health care processes.

	Title	Abbreviated Title	Year of Publication	Organisation	Region (Continent/Country/Autonomous Community)	Evidence Analysis for Quality Indicators (QIs)	Specific Breast Cancer Document	Subsection with Specific Information on Breast Cancer	Appearance of Quality Indicators (QIs)
1	Quality indicators in breast cancer care: An update from the EUSOMA working group.	EUSOMA	2017	EUSOMA	Europe	Review, consensus	Yes	Not applicable	Yes
2	Evaluación de la práctica asistencial oncológica. Estrategia en Cáncer del Sistema Nacional de Salud.	Spanish program	2013	Sistema Nacional de Salud	Spain	Consensus	No	Yes	Yes
3	Proceso Asistencial Integrado Cáncer de Mama (PAICM).	Andalusia	2011	Junta de Andalucía	Europe/Spain/Andalucía	Review	Yes	Not applicable	Yes
4	Proceso de Cáncer de Mama. Criterios de implantación.	Aragon	2006	Sistema de Salud de Aragón	Europe/Spain/Aragón	Consensus	Yes	Not applicable	Yes
5	Programas clave de Atención Interdisciplinar.	Asturias	2019	Gobierno del Principado de Asturias	Europe/Spain/Asturias	Not applicable	No	No	No
6	Estrategia regional del paciente oncologico en Castilla y León.	Castile and Leon	2019	Junta de Castilla y León	Europe/Spain/Castile and Leon	Review	No	No	Yes
7	Desarrollo de indicadores de proceso y resultado, y evaluación de la práctica asistencial oncológica.	Catalonia	2006	Generalitat de Catalunya	Europe/Spain/Catalonia	Review, consensus	No	Yes	Yes
8	Plan integral contra el cáncer en Extremadura.	Extremadura	2017	Junta de Extremadura	Europe/Spain/Extremadura	Not applicable	No	No	No
9	Proceso asistencial integrado de cancer de mama.	Galicia	2014	Xunta de Galicia	Europe/Spain/Galicia	Not specified	Yes	Not applicable	Yes
10	III plan de Salud La Rioja (2015–2019).	Rioja	2015	Gobierno de La Rioja	Europe/Spain/La Rioja	Based on the Nation Plan of Healthcare	No	No	Yes
11	Plan integral de control del cáncer de la Comunidad de Madrid.	Madrid	2007	Comunidad de Madrid	Europe/Spain/Madrid	Not applicable	No	No	No
12	Esta garantizada la calidad de la atención al cancer de mama.	Murcia	2012	Región de Murcia	Europe/Spain/Murcia	Based on the Nation Plan of Healthcare	Yes	Not applicable	Yes
13	Plan de Salud de Navarra.	Navarra	2014	Gobierno de Navarra	Europe/Spain/Navarra	Not applicable	No	No	No
14	Plan oncológico de Euskadi.	Basque country	2018	Gobierno Vasco	Europe/Spain/Basque Country	Not applicable	No	No	No
15	Estrategia contra el cancer de la Comunitat Valenciana 2019–2022.	Valencia	2019	Generalitat Valenciana	Europe/Spain/Valencia	Not applicable	No	No	No

## Data Availability

All the supplementary materials can be accessed upon request via email to the corresponding authors of this study.

## Abbreviations

BC: breast cancer, BCT: breast-conserving therapy, EUSOMA: European Society of Breast Cancer Specialists, ICC: intraclass coefficient, MDT: multidisciplinary team, MRI: magnetic resonance imaging, NANDA: North American Nursing Diagnosis Association, NS: not specified, PRISMA: Preferred Reporting Items for Systematic Reviews and Meta-Analyses, PST: primary systemic treatment, QIs: quality indicators, RT: radiotherapy, SDM: shared decision making, SLNB: sentinel lymph node biopsy.

## Appendix A. Data Sources and Search Strategy

### Appendix A.1. Sample Search Strategy for Medline

A systematic search was conducted on 19 February 2021 in Medline (via PubMed, to February 2021) using the following combination of free-text terms:

#1 breast cancer (all)
#2 breast neoplasms (all)
#3 quality indicators (all)
#4 quality care (all)
#5 2010 (pdta): 3000 (pdta)
# #6 AND #10 AND #11 AND #12
Results: 7 articles

### Appendix A.2. Online Databases

1. Embase
2. Scopus
3. Web of Science
4. Medline

### Appendix A.3. Websites of European Institutions

1. EUSOMA, Europe
2. Professional institutions and societies or governmental agencies from Spain

## References

[1] Loberg M., Lousdal M.L., Bretthauer M., Kalager M. (2015). Benefits and harms of mammography screening. Breast Cancer Res..

[2] Biganzoli L., Marotti L., Hart C.D., Cataliotti L., Cutuli B., Kühn T., Mansel R.E., Ponti A., Poortmans P., Regitnig P. (2017). Quality indicators in breast cancer care: An update from the EUSOMA working group. Eur. J. Cancer.

[3] Camps C.A.J., Antón A., Aranda E., Carrato A., Cassinello J. (2016). Quality indicators to assure and improve cancer care in Spain using the Delphi technique. J. Natl. Compr. Cancer Netw..

[4] Stordeur S., Vrijens F., Devriese S., Beirens K., Van Eycken E., Vlayen J. (2012). Developing and measuring a set of process and outcome indicators for breast cancer. Breast.

[5] van Hoeve J., de Munck L., Otter R., de Vries J., Siesling S. (2014). Quality improvement by implementing an integrated oncological care pathway for breast cancer patients. Breast.

[6] Donabedian A. (1966). Evaluating the quality of medical care. Milbank Meml. Fund Q..

[7] De Kok M.S.R., Sixma H.J., Van der Weijden T., Spijkers K.F., Van de Velde C.J. (2007). The patient’s perspective of the quality of breast cancer care. The development of an instrument to measure quality of care through focus groups and concept mapping with breast cancer patients. Eur. J. Cancer.

[8] AHRQ (2021). Tutorial on Quality Measures. http://qualitymeasures.ahrq.gov/tutorial/varieties.

[9] Elwyn G., Frosch D.L., Kobrin S. (2016). Implementing shared decision-making: Consider all the consequences. Implement. Sci..

[10] Maes-Carballo M., Martin-Diaz M., Mignini L., Khan K.S., Trigueros R., Bueno-Cavanillas A. (2021). Evaluation of the Use of Shared Decision Making in Breast Cancer: International Survey. Int. J. Environ. Res. Public Health.

[11] Veroff D., Marr A., Wennberg D.E. (2013). Enhanced support for shared decision making reduced costs of care for patients with preference-sensitive conditions. Health Aff..

[12] Elwyn G., Frosch D., Thomson R., Joseph-Williams N., Lloyd A., Kinnersley P., Cording E., Tomson D., Dodd C., Rollnick S. (2012). Shared Decision Making: A Model for Clinical Practice. J. Gen. Intern. Med..

[13] Perestelo-Perez L., Rivero-Santana A., Perez-Ramos J., Gonzalez-Lorenzo M., Roman J.G., Serrano-Aguilar P. (2011). Shared decision making in Spain: Current state and future perspectives. Z. Evid. Fortbild. Qual. Gesundhwes..

[14] Scheibler F., Stoffel M.P., Barth C., Kuch C., Steffen P., Baldamus C.A., Pfaff H. (2005). Shared decision-making as a new quality indicator in nephrology: A nationwide survey in Germany. Med. Klin..

[15] Tresserra F.A.C., Montealegre P., Martínez M.A., Fábregas R., Pascual M.A. (2017). Indicadores de calidad en el diagnóstico y tratamiento del cáncer para unidades de mama: Encuesta nacional. Rev. Senol. Patol. Mamar..

[16] Centro de Estudios Financieros (2021). Gestión Sanitaria. Sistema Sanitario Español Actual..

[17] Bao H., Yang F., Wang X., Su S., Liu D., Fu R., Zhang H., Liu M. (2015). Developing a set of quality indicators for breast cancer care in China. Int. J. Qual. Health Care.

[18] Nietz S., Ruff P., Chen W.C., O’Neil D.S., Norris S.A. (2020). Quality indicators for the diagnosis and surgical management of breast cancer in South Africa. Breast.

[19] Camejo N.C.C., Richter L., Artagaveytia N., Hernández A.L., Castro T., Castillo A., Sciuto P., Laviña G., Bernachin J., Milans S. (2015). Evaluación de la calidad de la asistencia en la Unidad Docente Asistencial de Mastología del Hospital de Clínicas. Rev. Méd. Urug..

[20] Cowppli-Bony A., Trétarre B., Marrer E., Defossez G., Daubisse-Marliac L., Coureau G., Minicozzi P., Woronoff A.S., Delafosse P., Molinié F. (2019). Compliance with clinical guidelines for breast cancer management: A population-based study of quality-of-care indicators in France. PLoS ONE.

[21] Donoso A.M., Minassian M., Salazaar C., Arbulo D., Slater J., Schwartz R., Gómez S., Kuen L. (2013). Indicadores de calidad en cáncer de mama. Rev. Chil. Cirugía.

[22] Ferrua M., Couralet M., Nitenberg G., Morin S., Serin D., Minvielle E. (2012). Development and feasibility of a set of quality indicators relative to the timeliness and organisation of care for new breast cancer patients undergoing surgery. BMC Health Serv. Res..

[23] Gobierno de España. Ministerio de Salud, Política Social e Igualdad (2010). Plan de Calidad para el Sistema Nacional de Salud. https://www.mscbs.gob.es/organizacion/sns/planCalidadSNS/.

[24] Moher D., Liberati A., Tetzlaff J., Altman D.G., Prisma Group (2009). Preferred reporting items for systematic reviews and meta-analyses: The PRISMA Statement. Open Med..

[25] Liberati A., Altman D.G., Tetzlaff J., Mulrow C., Gøtzsche P.C., Ioannidis J.P., Clarke M., Devereaux P.J., Kleijnen J., Moher D. (2009). The PRISMA statement for reporting systematic reviews and meta-analyses of studies that evaluate health care interventions: Explanation and elaboration. Ann. Intern. Med..

[26] Kinsman L., Rotter T., James E., Snow P., Willis J. (2010). What is a clinical pathway? Development of a definition to inform the debate. BMC Med..

[27] SESPM (Spanish Foundation of Senology and Breast Disease) (2020). Breast Cancer Clinical Pathway. https://www.sespm.es/wp-content/uploads/2020/07/LIBRO-VIA-CLINICA-SESPM-2020-ingles-patro.pdf.

[28] Koo T.K., Li M.Y. (2016). A Guideline of Selecting and Reporting Intraclass Correlation Coefficients for Reliability Research. J. Chiropr. Med..

[29] Sistema Nacional de Salud (2013). Evaluación de la Práctica Asistencial Oncológica. Estrategia en Cáncer del Sistema Nacional de Salud. http://saludcantabria.es/uploads/pdf/noticias/Punto%2011-%20Evaluaci%C3%B3n%20de%20la%20pr%C3%A1ctica%20asistencial%20oncol%C3%B3gica.pdf.

[30] Acebal Blanco M.M., Alba Conejo E., Álvarez Benito M., Bayo Lozano E., Del Río Urenda S., Dotor Gracia M., Dueñas Rodríguez B., Fernández Echegaray R., Fernández Temprano J.M., Galván Ruiz A. (2011). Cáncer de Mama: Proceso Asistencial Integrado.

[31] Sistema de Salud de Aragón (2006). Proceso de Cáncer de Mama. Criterios de Implantación. https://www.aragon.es/documents/20127/674325/Implantacion%20proceso%20CANCER%20DE%20MAMA.pdf/d97120bb-72c5-8997-6a8b-8cf516c69faf.

[32] Gobierno del Principado de Asturias (2019). Programas Clave de Atención Interdisciplinar. https://www.astursalud.es/noticias/-/noticias/pcai-cancer-de-mama.

[33] Junta de Castilla y León (2019). Estrategia Regional del Paciente Oncologico en Castilla y León. https://www.saludcastillayleon.es/institucion/es/atencion-paciente-oncologico.ficheros/1308260-Estrategia%20ONCYL%20%28P%C3%A1ginas%20completo%29.pdf.

[34] Generalitat de Catalunya (2006). Desarrollo de Indicadores de Proceso y Resultado, y Evaluación de la Práctica Asistencial Oncológica. https://www.sergas.es/Docs/Avalia-t/AATRM200602.pdf.

[35] Junta de Extremadura (2017). Plan Integral Contra el Cáncer en Extremadura. https://saludextremadura.ses.es/filescms/web/uploaded_files/CustomContentResources/PLAN%20INTEGRAL%20CONTRA%20EL%20CANCER%202017-2021.pdf.

[36] Xunta de Galicia (2014). Proceso Asistencial Integrado de Cancer de Mama. https://www.sergas.es/Asistencia-sanitaria/Proceso%20asistencial%20integrado%20de%20cancro%20de%20mama.

[37] Gobierno de La Rioja (2015). III Plan de Salud La Rioja (2015–2019). https://www.riojasalud.es/files/content/ciudadanos/planes-estrategicos/III-plan-salud.pdf.

[38] Comunidad de Madrid (2007). Plan Integral de Control del Cáncer de la Comunidad de Madrid. http://www.madrid.org/bvirtual/BVCM009363.pdf.

[39] Región de Murcia (2012). ¿Esta Garantizada la Calidad de la Atención al Cancer de Mama?. https://sms.carm.es/somosmas/documents/63024/125910/JOSEBA+C%C3%A1ncer+de+mama+Bilbao+2012.pdf/f74d7d5f-64f5-422d-9614-6c7ac3634436.

[40] Gobierno de Navarra (2014). Plan de Salud de Navarra. http://www.navarra.es/home_es/Temas/Portal+de+la+Salud/Ciudadania/Nuevo+Modelo+asistencial/Plan+Salud+Navarra/Plan+de+Salud+de+Navarra+2014-2020+Profesionales/.

[41] Gobierno Vasco (2018). Plan oncológico de Euskadi. https://www.euskadi.eus/contenidos/informacion/plan_oncologico_2018_2023/es_def/adjuntos/plan-oncologico-euskadi-2018-2023.pdf.

[42] Generalitat Valenciana (2019). Estrategia contra el cancer de la Comunitat Valenciana 2019–2022. https://socvalped.com/wp-content/uploads/2019/06/EstrategiaCancer20190121.pdf.

[43] Fadda J., Sayigh A. (2019). Quality of Healthcare: A Review of the Impact of the Hospital Physical Environment on Improving Quality of Care: Selected Papers from the World Renewable Energy Network’s Med Green Forum 2017. Sustainable Building for a Cleaner Environment. Innovative Renewable Energy.

[44] Hewitt M., Simone J.V., Malin J., Kahn K.L., McGlynn E.A., Asch S., Keesey J., Hicks J., DeCristofaro A.H., Kerr E. (2006). Measuring the Quality of Cancer Care. The National Initiative for Cancer Care Quality (NICCQ).

[45] Albert J.M., Das P. (2012). Quality assessment in oncology. Int. J. Radiat. Oncol. Biol. Phys..

[46] Yu K.D., Fan L., Qiu L.X., Ling H., Jiang Y.Z., Shao Z.M. (2017). Influence of delayed initiation of adjuvant chemotherapy on breast cancer survival is subtype-dependent. Oncotarget.

[47] Kumar P.A., Wang D., Sinha S., Sivapiragasam A. (2019). Does the time to initiate adjuvant chemotherapy effect outcome in patients with breast cancer? A National Cancer Database-based retrospective analysis. J. Clin. Oncol..

